# Morbidity and Mortality Analysis in the Treatment of Intertrochanteric Hip Fracture with Two Fixation Systems: Dynamic Hip Screw (DHS) or Trochanteric Fixation Nail Advance (TFNA)

**DOI:** 10.3390/geriatrics8030066

**Published:** 2023-06-08

**Authors:** Alvaro López-Hualda, Elsa Arruti-Pérez, Fátima N. Bebea-Zamorano, María Dolores Sosa-Reina, Jorge Hugo Villafañe, Javier Martínez-Martin

**Affiliations:** 1 Orthopedic Surgery and Traumatology Service, Hospital Universitario Fundación Alcorcon, 28922 Alcorcón, Spain; elsa.arruti@salud.madrid.org (E.A.-P.); fatimangole.bebea@salud.madrid.org (F.N.B.-Z.);; 2 Department of Physiotherapy, Faculty of Sports Sciences, Universidad Europea de Madrid, Calle Tajo s/n, 28670 Villaviciosa de Odón, Spain; mariadolores.sosa@universidadeuropea.es; 3 Musculoskeletal Pain and Motor Control Research Group, Faculty of Sport Sciences, Universidad Europea de Madrid, 28670 Villaviciosa de Odón, Spain; 4 IRCCS Fondazione Don Carlo Gnocchi, 20148 Milan, Italy

**Keywords:** complications, hip fracture, surgery

## Abstract

Background: The aim of this study was to compare the clinical outcomes, complications, and mortality of patients with intertrochanteric hip fracture treated with dynamic hip screw (DHS) vs. trochanteric fixation nail advance (TFNA). Methods: We evaluated 152 patients with intertrochanteric fractures concerning age, sex, comorbidity, Charlson Index, preoperative gait, OTA/AO classification, time from fracture to surgery, blood loss, amount of blood replacement, changes in gait, full weight-bearing at hospital discharge, complications, and mortality. The final indicators encompassed the adverse effects linked to implants, postoperative complications, clinical healing or bone healing duration, and functional score. Results: The study included a total of 152 patients, out of which 78 (51%) received DHS treatment and 74 (49%) received TFNA treatment. The results of this study show that the TFNA group demonstrated superiority (*p* < 0.001). However, it should be noted that the TFNA group had a higher frequency of the most unstable fractures (AO 31 A3, *p* < 0.005). Full weight-bearing at discharge also decreased in patients with more unstable fractures (*p* = 0.005) and severe dementia (*p* = 0.027). Mortality was higher in the DHS group; however, a longer time from diagnosis to surgery was also observed in this group (*p* < 0.005). Conclusions: The TFNA group has shown a higher success rate in achieving full weight-bearing at hospital discharge when treating trochanteric hip fractures. This makes it the preferred choice for treating unstable fractures in this region of the hip. Additionally, it is important to note that a longer time to surgery is associated with increased mortality in patients with hip fractures.

## 1. Introduction

Hip fractures represent a significant and growing public health concern worldwide. In Spain specifically, the incidence of hip fractures is reported to be around 104 cases per 100,000 inhabitants. This translates to approximately 45,000 to 50,000 hip fractures occurring each year in the country [1]. The mortality rate associated with hip fractures is an important aspect to consider. Studies have indicated that there is a mortality rate of 7.1% directly related to the fracture itself. This means that a notable number of individuals who experience a hip fracture also face an increased risk of death as a result. It is crucial to address this issue and implement effective strategies to improve outcomes and reduce mortality rates. Therefore, it is crucial to implement prevention strategies promptly. Fortunately, there are now well-validated methods available for detecting the risk of proximal femoral fractures, including clinical risk-factor assessment and various imaging and biochemical assessments such as CT scans, DEXA, US, and bone fragility analysis. These methods can aid in the early identification and prevention of these fractures [2].

Hip joint replacement, also known as total hip arthroplasty, is a surgical procedure that involves the physical replacement of a damaged or diseased human hip joint with a hip joint prosthesis [3]. This procedure is commonly performed to alleviate pain, improve joint function, and enhance the quality of life for patients with severe hip joint conditions [4]. It is important to perform interventions with the least aggressive surgical techniques, as well as to use implants that allow early ambulation and full weight-bearing. The aim of this study was to avoid complications derived from long-term bed rest (atrophy, pressure ulcers, and respiratory infection), which is the main cause of death in the first year. This process implies high and increasing economic expenditures. The average expenditure per patient during the first year after admission has been calculated at approximately EUR 12,000, and the hospital admission itself costs EUR 4740.29 [5]. The socio–economic impact of hip fractures is significant, driving substantial advancements in the field of osteosynthesis. These developments have focused on improving fracture management, enhancing implant stability, and promoting early mobilization. By reducing healthcare costs and improving patient outcomes, these advancements contribute to the overall management and mitigation of the socio–economic burden associated with hip fractures.

The dynamic hip screw (DHS) technique, a commonly used method, affects postoperative early ambulation in patients due to the unsatisfactory stability of the internal fixation in unstable fractures [6,7,8]. Despite the simplicity of this surgical technique, significant damage to the muscle can occur. Recently, proximal femoral nails represent a minimally invasive alternative for fixation, reducing soft tissue injury and achieving greater biomechanical stability [9,10,11,12,13]. The trochanteric fixation nail advance (TFNA) is a new technique that presents theoretical advantages that allow stable fixation of the most unstable fractures. The TFNA implant has been shown to have good clinical outcomes in the management of unstable trochanteric fractures. Studies have demonstrated its effectiveness in achieving fracture reduction, maintaining stability, and facilitating early mobilization. Additionally, the TFNA has shown a low rate of complications such as implant failure, infection, and nonunion. Only a few articles have been published on the follow-up of this implant [14]; therefore, it is particularly important to carry out studies that compare the results with other implants. Over the past three decades, researchers have utilized walking conditions as a means to study load-bearing behavior in different domains, such as solid and fluid. However, due to the computational complexity involved, several studies have resorted to simplifications in walking conditions, which can potentially impact the results and lead to misinterpretation [15].

Hip fractures represent a significant public health problem in Spain, with a high incidence and associated mortality rate. Addressing this issue requires comprehensive strategies focused on prevention, timely treatment, and multidisciplinary care. By prioritizing effective interventions and collaborative efforts, the aim is to improve outcomes and enhance the overall well-being of individuals affected by hip fractures. The main objective of this study was to compare the clinical functional outcomes, complications, and mortality between two intertrochanteric hip fracture fixation systems: the DHS and the TFNA. As a secondary objective, the study also analyzed other factors that could potentially influence morbidity, mortality, and functional recovery following the fracture.

## 2. Materials and Methods

A retrospective cohort study was conducted from January 2016 to December 2018 in patients with intertrochanteric hip fractures. Surgical fixation was performed using a dynamic hip screw Omega2 (Stryker, Kalamazoo, MI, USA) [EO2] or a proximal femoral nail TFNA (Depuy-Synthes, Warsaw, IN, USA). Exclusion criteria included patients aged less than 60 years, pathological fractures, multiple fractures, a subtrochanteric extension of the fracture trace, and follow-up time below radiological consolidation.

A total of 152 patients were included in the study, of which 78 and 74 patients were treated with DHS and TFNA, respectively.

The variables were divided into two categories: preoperative and postoperative. Preoperative variables included age, sex, medical comorbidities, Charlson Index, treatment with anticoagulants and/or anti-aggregates, for the classification of pertrochanteric fractures, the AO Foundation/Orthopaedic Trauma Association (AO/OTA) combined fracture classification system is widely accepted and discussed, preoperative gait, preoperative hemoglobin (g/dL), and days from fracture diagnosis to surgery. Postoperative variables included postoperative hemoglobin, anemisation, blood transfusion, number of red blood cells, full weight-bearing at hospital discharge, postoperative complications (infection, mechanical failure, and cut-out), re-operation, gait at the end of follow-up, and mortality.

The study was approved by the ethics committee of the hospital, and informed consent was obtained from each patient.

### 2.1. Surgical Treatment

Patients were taken up for surgery as early as possible after relevant investigations, radiographs, and anesthetic clearance. All patients were operated on after spinal anesthesia and positioned on an orthopedic traction table in supine position. Assistance of intraoperative fluoroscopy is necessary to carry out the intervention. All patients undergo an intraoperative safety checklist and antibiotic prophylaxis (cefazolin 1 g) prior to making the incision. The implant (DHS or TFNA) was chosen according to the preference of the main surgeon.

DHS. Surgery is performed through an 8 cm approach in the lateral side of the hip distal to the greater trochanter. Opening the fascia lata and raising the vastus lateralis muscle is mandatory to place a DHS. The Omega 2 (Stryker) is a dynamic hip screw (DHS) which includes a dynamic compression plate with 135° cervical angulation and 4 holes. A cervical lag screw should be centered in the head on both anterior–posterior and lateral views. Afterwards, four cortical 4.5 screws apply the plate against the lateral cortex of the femur.

TFNA. A 5 cm longitudinal incision is made proximal to the greater trochanter; muscle fibers of the gluteus medius are then divided to access the greater trochanter. A needle is placed at the entry point, which would serve as a guide for reaming the medullary canal. The appropriate TFNA nail (Depuy Synthes) is inserted into the canal. A helical blade is placed through the nail to the femoral head; placement should be monitored using fluoroscopy. Finally, the system is locked with a 5 mm distal screw. The TFNA Nail is anatomically contoured to a nominal diameter of 9–10 mm and short lengths of 170–235 mm. The proximal locking hole accommodates angles ranging from 125° to 130°.

After the surgery, all patients received thromboprophylaxis. Blood tests and postoperative X-ray were performed, and patients underwent regular physiotherapy and orthogeriatric review. Early seating and active mobilization of the operated limb started within the first 24 h. Full weight-bearing was agreed upon once the complexity of the fracture and the fixation achieved were assessed.

Clinical and radiological follow-up was performed at four weeks, three months, and six months after surgery.

### 2.2. Data Collection and Statistical Analysis

Data were assessed statistically using SPSS Statistics software (version 26.0), USA. The qualitative variables were presented as absolute frequencies and percentages and were compared with the Pearson/Fisher exact test χ^2^. Quantitative variables were described as mean ± standard deviation. Quantitative data distributions were checked for normality using the Shapiro–Wilk normality test, and these variables were compared with the Student’s t-test. If laws of normality were not followed, they were compared with the non-parametric Mann–Whitney U-range test. Quantitative variables in more than two groups were compared with the analysis of variance (ANOVA) test. If the conditions for applying this test (normality of data and homogeneity) were not met, the non-parametric Kruskal–Wallis test was used. Statistical significance was set at *p* < 0.05. The alpha error was set at 0.05, and the statistical power was at 0.80.

## 3. Results

### 3.1. Demographic Distribution

A total of 152 patients were included, of whom 78 (51%) were treated with DHS and 74 (49%) were treated with TFNA. No differences in age, sex, or previous comorbidities were found. The majority of patients were female (82.9%, *n* = 126). The mean age was 84.4 ± 7.2 years, Table 1.

Stable fractures were more common in A1 (*n* = 72, 47%) and A2 (*n* = 68, 45%) categories compared to unstable fractures (A3, *n* = 12, 8%). The distribution of fractures was similar between the two groups, except for the most unstable fractures (A3), where the TFNA was preferred in 10 patients compared to the Dynamic Hip Screw (DHS) used in two patients (*p* = 0.035). Both groups had similar preoperative comorbidities, which were studied independently and using the Charlson Index. The most frequent were arterial hypertension (68.4%), chronic cardiopathy (39.5%), and diabetes mellitus (32.2%). Severe dementia was observed in 29.8% of patients. It was recorded that 32.9% of patients were anti-aggregated and 17.8% were anticoagulated preoperatively.

The average time from fracture to surgery was 3.3 ± 2.5 days. Differences were observed between both groups (TFNA and DHS), being 2.74 ± 1.1 days and 4.18 ± 1.8 days on average, respectively.

Patients taking oral anticoagulants have been shown to have a longer delay in performing surgery, with an average delay of 5.4 ± 1.9 days. However, this has not been observed in anti-aggregate patients.

It was also found that patients with a Charlson Index of ≥2 points were operated on significantly later (*p* = 0.002), with a mean delay of 2.1 ± 2.1 days.

### 3.2. Blood Loss and Transfusions

No differences were found between the groups in preoperative and postoperative hemoglobin levels (*p* > 0.05).

The mean values for preoperative hemoglobin, postoperative hemoglobin, and anemisation were 12.31 ± 2 g/dL, 10.03 ± 1.63 g/dL, and 2.86 ± 1.26 g/dL, respectively.

Concerning the need for transfusion of patients who had been anemized, 35 patients (23%) required blood transfusion, 9 patients received one red blood cell (RBC) concentrate, 19 patients received two RBC concentrates, and seven patients received three or more RBC concentrates.

No differences were found in anemization or transfusion between the study groups (*p* = 0.711), Table 2.

More statistically significant anemization was observed according to age, type of fracture, and previous treatment with anti-aggregates.

Greater anemization was found at a higher age (*p* = 0.056). The highest figures are in the group over 90 years old, with a mean of 3.839 ± 1.32 g/dL.

Blood loss was higher in patients with more unstable fractures (*p* = 0.03). Fractures 31A2, 31A3, and 31A1 had a mean of 2.95 ± 1.33 g/dL, 3.8 ± 1.5 g/dL, and 2.6 ± 1.05 g/dL, respectively.

It was also observed that patients who received anti-aggregate treatment had a greater drop in hemoglobin (3.1 ± 1.23 g/dL) than those who were not anti-aggregated (2.52 ± 1.28 g/dL) (*p* = 0.023). No significant differences were observed in anticoagulated patients (*p* = 0.544).

Regarding postoperative transfusion, only female sex and previous hip fracture were identified as factors that increased the risk of transfusion (*p* = 0.034). This is justified because these patients start from a situation of anemia before the fracture.

### 3.3. Post-Operative Mechanical Complications

Of the 152 operations performed, seven post-surgical complications (4.6%) were recorded, five dismantling of the patients treated with DHS, and two cut-outs in patients operated with TFNA.

No significant differences in complications were observed concerning the type of implant used (*p* = 0.1470) or the type of fracture (*p* = 0.147).

It was observed that patients who were functionally more autonomous (independent gait or with support) presented fewer complications than those who departed with a more deteriorated pre-fracture gait (*p* = 0.041). The remaining variables did not show any differences in the analysis.

### 3.4. Functional Outcomes and Weight-Bearing

In both groups, deterioration of the march was observed concerning the situation before the fracture, with an increase in dependency (*p* = 0.003), Table 3.

No differences in previous gait (*p* = 0.095) or worsening of gait (*p* = 0.120) were observed between the two groups; however, there was a significant difference in the burden of the operated limb at hospital discharge.

In the DHS group, 44.9% of the patients carried out load at discharge; however, in the TFNA group, it reached 67.6% (*p* = 0.005), even though the most unstable fractures were more frequent in this group (A3), Table 2.

Among all the variables, only vascular pathology was shown to be a relevant factor in the deterioration of walking (*p* = 0.017).

The burden at hospital discharge decreased significantly in patients with more unstable fractures (*p* = 0.005), fracture fixation with extramedullary devices (*p* = 0.005), and severe cognitive impairment (*p* = 0.027).

On the other hand, it has been shown that patients who have a better gait before the fracture (independent or with support) show a lower rate of mechanical complications (*p* = 0.04), higher probability of load at hospital discharge (*p* = 0.001), and lower need for transfusion (*p* = 0.002).

### 3.5. Mortality

A total of 17 patients died during follow-up, representing an overall mortality rate of 11.2% in both groups. Sixteen patients belonged to the DHS group, which means a 15.18 times higher mortality rate in this group (risk ratio: 15.18, 95% confidence interval [2.06–111.16]).

Delaying the surgery from the time of diagnosis increased the mortality rate, and from the total number of deaths, there was an increase in the delay of surgery of >2 days in 13 patients (*p* = 0.048).

However, this was not influenced by the type of fracture, previous comorbidities, blood loss, transfusion, and post-surgical mechanical complications (*p* = 0.177).

## 4. Discussion

The study aimed to compare the effectiveness of the DHS and TFNA fixation systems in terms of clinical functional outcomes, complications, and mortality. Additionally, it sought to explore other factors that might play a role in patient recovery and prognosis following intertrochanteric hip fractures. The study findings indicate that the TFNA group had a higher success rate in achieving full weight-bearing at hospital discharge when treating trochanteric hip fractures. Therefore, the TFNA implant is recommended for managing the most unstable fractures in this region. Furthermore, the study revealed that there is an increase in mortality among patients who experience a longer time for surgery. Hence, patients with hip fractures must undergo surgery within two days of admission to optimize outcomes and reduce mortality risks. The results of this study could contribute to informing clinical decision-making and improving patient care in the treatment of these fractures. In their study, Zhang et al. [13] investigated the postoperative femoral fracture rates following the removal of implants such as DHS or PFNA. Surprisingly, they did not observe an increase in femoral fractures or detect any significant differences between the two implant types. Furthermore, their research did not establish a time-dependent change for either implant.

The socio–economic impact of hip fractures arises from various factors. First and foremost, the direct healthcare costs associated with hip fractures include expenses related to hospitalization, surgical procedures, postoperative care, rehabilitation, and long-term management. Additionally, indirect costs arise from lost productivity, decreased independence, increased need for assistance, and potential long-term care requirements. The overall economic impact is substantial, affecting not only individuals but also healthcare systems, insurers, and society as a whole [16]. During the last few decades, this pathology has led to an increase in the consumption of resources. The optimal device to ensure early loading and ambulation with the least possible aggressive surgical technique is still debated and controversial [17,18].

The sliding plate screw is an extramedullary fixation system and has classically been considered the gold standard [19]. This intervention, although technically simple, requires the dissection of soft parts that can cause considerable bleeding, greater postoperative pain, and complications that can hinder postoperative walking [9,12,19,20,21,22,23,24]. Over the past 30 years, research on artificial hip joint bearings using computational simulation has often relied on simplified walking conditions, particularly in terms of loading, motions, and cycle components. However, to obtain more realistic results that accurately reflect the physiological behavior of the human hip joint during walking, future studies should aim to eliminate simplifications [15]. The main concern in total hip arthroplasty (THA) is wear, which plays a crucial role in the deterioration of the joint and the release of debris into the body. The wear process occurs during the running-in phase, which is the initial stage where two surfaces interact before reaching a steady-state phase. Understanding the running-in phase is essential for comprehending hip joint wear [25]. The findings reveal that impingement is likely to occur during the transition from standing to prostration, during the transition phase, and while sitting. These particular positions and movements show a higher risk of impingement in individuals who have undergone THA [26].

While the DHS may be a viable option for certain subtrochanteric fractures, it is important to note that the DHS system has shown worse results in treating unstable fractures and intertrochanteric fractures with fractures of the lateral cortex. These types of fractures pose specific challenges and are associated with higher risks of complications when treated with DHS. One concern with using DHS for unstable subtrochanteric fractures is the reported high rates of femoral medial displacement, non-union (the failure of fractured bones to heal properly), and subsequent re-operation. These issues have raised doubts about the effectiveness of DHS in these specific cases [27]. However, despite these concerns, the DHS remains a fixation method for subtrochanteric fractures, and some surgeons continue to use it. One advantage of the DHS is its ability to provide compression along the femoral neck, which can aid in promoting healing. Additionally, the DHS allows for load sharing between the bone and the implant, which is beneficial for certain fracture types [28]. Occasionally, fracture of the lateral cortex can occur iatrogenically with the broaching of the head screw in very narrow lateral cortices and in patients with small femurs [29]. Experimental biomechanical studies, such as those conducted by Weiser et al., concluded that the load required for fracture disassembly was significantly lower with the extramedullary device [30]. Endomedullary devices are designed to reduce the lever arm, which helps in transmitting forces along the physiological axis of the femur. This design feature increases the load required for mechanical failure. However, one characteristic mechanical complication associated with endomedullary nails is “cut-out”. Cut-out refers to a specific type of failure where the screw or implant used in the procedure cuts or penetrates through the bone, leading to loss of fixation and potential instability [31].

The present study confirms that the characteristic complications of the DHS implant were the mechanical failure, while in the TFNA it was the “cut-out”. In addition, the most unstable fractures were frequently treated with endomedullary nailing, a fact that coincides with that reported in the literature [9,21,32,33]. Many authors have extended this statement to stable fractures, stating that there are fewer complications in AO 31 A1 and A2 fractures [34,35,36]. The group treated with TFNA had the highest percentage of patients who achieved a march with the support of the operated member at discharge. This fact is especially relevant since early walking improves later functional evolution and reduces the risk of complications associated with hip fractures. Furthermore, it constitutes the main objective of hip fracture surgery. The TFNA is indeed a widely accepted implant used for the treatment of unstable fractures, specifically those involving the trochanteric region of the femur. The TFNA is designed to provide stability and support to the fractured bone, allowing for proper healing and restoration of function.

We agree with the result published by Duymus et al. that the study suggests that the new generation intra-medullary nails are preferred over extra-medullary implants for the treatment of A2 unstable intertrochanteric femur fractures (IFFs). These intra-medullary nails are easier to apply and have shown more successful clinical outcomes [37]. However, in cases where PF-LCP is used, it is crucial to ensure that early weight-bearing is avoided until the formation of callus is observed. This caution is necessary to prevent complications and promote proper healing of the fracture. Most articles state that intramedullary devices are superior in terms of functional results [9,13,20,21]. The researchers [35,36] found that the rate of reoperations, primarily due to femoral fractures after implant removal, was higher in patients who underwent DHS compared to PFNA. They also observed clinically significant differences in postoperative Harris Hip Score (HHS) after one year of follow-up, with poorer performance in the DHS group. Although the study had some limitations, the results were consistent with certain meta-analyses of RCTs. While there seemed to be a trend favoring DHS as the preferred treatment for stable IFFs, this study indicated a higher rate of radiographic complications associated with DHS compared to PFNA fixation among elderly patients with osteoporotic type 31-A1 IFFs.

Recent publications have found a greater postoperative anemization in interventions where extramedullary systems were used, which may be due to the dissection of the lateral thigh muscles required in the intervention [9,12,20,21,22]. According to Li et al., the observational group demonstrated significant improvements compared to the control group in several aspects. The operational duration, hemorrhaging, and drainage volume were all reduced in the observational group (*p* < 0.05) [21]. However, other authors agree with our results, in which we did not find differences in the anemization figures or the transfusion rate [10,13]. Careful technique and thorough hemostasis can reduce bleeding in the DHS technique. By minimizing bleeding, surgeons can improve visibility, reduce the risk of complications associated with excessive bleeding, and enhance the overall success and safety of the DHS procedure.

In the study population, postoperative mortality was higher in the group that underwent the DHS procedure. However, it was observed that the time from diagnosis to intervention was also longer in this group. The multivariate analysis conducted in the study identified a delay in intervention as an independent risk factor for increased mortality. This finding justifies the differences observed between the two groups and is consistent with previous publications [38,39,40]. Most articles published on this topic have reported no significant differences in mortality based on the type of implant used [10,11,12,34,36,41]. However, one study by Whitehouse et al. [41] found that the use of intramedullary systems had a higher risk of mortality at 30 days postoperative compared to the group treated with an extramedullary system. It is important to interpret these results with caution as there may be other related factors that act as confounders. Recent studies have recommended further research on predictors of mortality about hip fractures. The study by Jantzen et al. [42] suggests that certain drugs can be predictive of higher mortality rates, indicating the need for intensified care for patients at increased risk. This underscores the importance of identifying and managing medication-related risks in hip fracture patients. Additionally, another study [43] conducted by researchers evaluates existing prediction models for mortality after hip fracture surgery. Lastly, the study conducted by Parker and Anand [44] investigates the causes of death and the time elapsed since injury to determine the true mortality rate associated with hip fractures. The study findings reveal a mortality rate of 15% specifically attributed to hip fractures, while the remaining deaths are attributed to age-related conditions. This highlights the impact of hip fractures on mortality and emphasizes the need for comprehensive care in managing both the fracture and associated age-related conditions.

According to the results obtained, it can be stated that the current trend is to use endomedullary fixation systems in the most unstable fractures, given that they present fewer mechanical complications and a higher percentage of patients who carry out early loading, the main objective in recovery [45,46,47,48,49,50]. This reduces the complications associated with prolonged bedding and may be a relevant factor in reducing the mortality associated with hip fractures. Another factor that has been shown to contribute to a decrease in mortality is a shortened delay from diagnosis to intervention [38,39,40,43,44,51].

Overall, the study highlights the importance of considering baseline comorbidities and their potential impact on surgical timing and patient outcomes in cases of hip fractures. It suggests that addressing chronic diseases and minimizing surgical delays may help reduce the risk of complications and improve the chances of successful recovery, particularly in older patients or those with pre-existing mobility issues, advanced dementia, or unstable fractures.

Although our study consistently revealed important insights from various orthopedic departments, it is essential to acknowledge several limitations. Firstly, the retrospective study design inherently carries drawbacks and limitations that can impact the reliability and validity of the findings. Secondly, there is a possibility of confounding factors related to both patients and surgeons that may have influenced the observed results, and these factors were not fully accounted for in the study. Additionally, being an observational study, there is a potential for overlooking certain confounding variables that could have affected the outcomes of interest. Furthermore, due to the retrospective nature of the study, there is a possibility that important variables contributing to complications were not adequately captured or analyzed. Lastly, the relatively short follow-up period limits our understanding of potential long-term complications associated with implants, which may manifest in the future.

## 5. Conclusions

In conclusion, the study compared the effectiveness of the DHS and TFNA fixation systems in treating trochanteric hip fractures. The results revealed that the TFNA group demonstrated higher success rates in achieving full weight-bearing at hospital discharge, suggesting it as the preferred choice for unstable fractures in this region. This information is relevant for orthopedic surgeons as they can make informed decisions based on factors such as the fracture pattern, bone quality, patient characteristics, and surgical expertise when selecting the appropriate implant.

Furthermore, the importance of timely surgery in managing hip fractures was emphasized, as it plays a crucial role in minimizing associated mortality rates. The results support the need for early intervention to optimize outcomes and reduce risks for patients.

Additionally, addressing baseline comorbidities and chronic diseases in the recovery process was highlighted. Older patients, those with mobility issues, advanced dementia, or unstable fractures may particularly benefit from comprehensive care that takes into account these underlying conditions. By considering these factors, healthcare professionals can reduce complications and improve long-term outcomes.

It is important to note that medical research is constantly evolving, and new advancements may emerge in the future that could influence the preferred choice of implant or surgical approach. Therefore, staying updated with ongoing research and consulting with medical professionals are essential for making evidence-based decisions.

Moreover, the socio–economic impact of hip fractures, including direct healthcare costs such as hospitalization, surgery, and rehabilitation, as well as indirect costs such as productivity loss and long-term care needs, was emphasized. These aspects underscore the importance of efficiently and effectively addressing hip fractures from both the patient’s perspective and the healthcare system and society as a whole.

## Figures and Tables

**Table 1:** Pre-operative variables.

Variables	TFNA (*n* = 74)	%	DHS (*n* = 78)	%	*p*-Value
Gender					
Female	59	79.7	67	85.9	0.313
Male	15	20.3	11	14.1	
Age					
<80 yrs	17	23.0	17	21.8	0.862
≥80 yrs	57	77.0	61	78.2	
Charlson Index					
0	39	52.7	37	48.1	0.677
1	21	28.4	27	35.1	
≥2	14	18.9	13	16.9	
AO classification					
31A1	31	41.9	41	52.6	0.035
31A2	33	44.6	35	44.9	
31A3	10	13.5	2	2.6	
Preoperative mobility					
Without aids 1	42	56.8	30	38.5	
crutch	18	24.3	21	26.9	0.095
2 crutches/walking frame	9	12.2	22	28.2	
Not walking	5	6.8	5	6.4	
Days to surgery	2.74		4.18		0.005

**Table 2:** Pre-operative variables.

Variables	TFNA (*n* = 74)	%	DHS (*n* = 78)	%	*p*-Value
Pre-operative hemoglobin (g/dL)	12.33		12.11	26.9	0.095
Anemization (g/dL)	2.89		2.82		0.711
Transfusion (*n*)	18	24.3	17	21.8	0.711
Complications (*n*)	2		5		1.0
Weight-bearing at hospital discharge (*n*)	50	67.6	35	44.9	0.005
Impairment of the gait (*n*)	49	66.2	42	53.8	0.120
Death (*n*)	1	1.4	16	20.5	<0.001

**Table 3:** Pre-operative gait and gait at the end of the follow-up.

	Pre-Fracture Gait	Gait after Follow-Up
Without support	72 (47.4%)	26 (17.1%)
One crutch	39 (25.7%)	28 (18.4%)
Two crutches	13 (8.6%)	11 (7.2%)
Orthopedic walker	18 (11.8%)	50 (32.9%)
Not walking	10 (6.6%)	37 (24.3%)

## Data Availability

Not applicable.
